# A *Schistosoma haematobium*-Specific Real-Time PCR for Diagnosis of Urogenital Schistosomiasis in Serum Samples of International Travelers and Migrants

**DOI:** 10.1371/journal.pntd.0002413

**Published:** 2013-08-29

**Authors:** Lieselotte Cnops, Patrick Soentjens, Jan Clerinx, Marjan Van Esbroeck

**Affiliations:** Department of Clinical Sciences, Institute of Tropical Medicine, Antwerp, Belgium; Centers for Disease Control and Prevention, United States of America

## Abstract

**Background:**

Diagnosis of urogenital schistosomiasis by microscopy and serological tests may be elusive in travelers due to low egg load and the absence of seroconversion upon arrival. There is need for a more sensitive diagnostic test. Therefore, we developed a real-time PCR targeting the *Schistosoma haematobium*-specific *Dra1* sequence.

**Methodology/Principal Findings:**

The PCR was evaluated on urine (n = 111), stool (n = 84) and serum samples (n = 135), and one biopsy from travelers and migrants with confirmed or suspected schistosomiasis. PCR revealed a positive result in 7/7 urine samples, 11/11 stool samples and 1/1 biopsy containing *S. haematobium* eggs as demonstrated by microscopy and in 22/23 serum samples from patients with a parasitological confirmed *S. haematobium* infection. *S. haematobium* DNA was additionally detected by PCR in 7 urine, 3 stool and 5 serum samples of patients suspected of having schistosomiasis without egg excretion in urine and feces. None of these suspected patients demonstrated other parasitic infections except one with *Blastocystis hominis* and *Entamoeba* cyst in a fecal sample. The PCR was negative in all stool samples containing *S. mansoni* eggs (n = 21) and in all serum samples of patients with a microscopically confirmed *S. mansoni* (n = 22), *Ascaris lumbricoides* (n = 1), *Ancylostomidae* (n = 1), *Strongyloides stercoralis* (n = 1) or *Trichuris trichuria* infection (n = 1). The PCR demonstrated a high specificity, reproducibility and analytical sensitivity (0.5 eggs per gram of feces).

**Conclusion/Significance:**

The real-time PCR targeting the *Dra1* sequence for *S. haematobium*-specific detection in urine, feces, and particularly serum, is a promising tool to confirm the diagnosis, also during the acute phase of urogenital schistosomiasis.

## Author Summary

Schistosomiasis is a disease caused by parasitic worms of the genus *Schistosoma*. About 200 million people are affected worldwide. Also travelers are at risk as even a brief contact with infested freshwater can cause infection. *S. mansoni* and *S. haematobium* are the two main species that are identified in travelers and migrants. The eggs of these parasites are respectively excreted in feces and urine, and the diagnosis relies mostly on microscopy. In travelers, infections are easily missed due to low worm load or because egg excretion is not yet started upon arrival. Consequently, there is need for sensitive diagnostic tools that can be used in the early stage of infection. A previously published study reported the ability to detect *S. mansoni* DNA in serum by real-time PCR. To enable the diagnosis of urogenital schistosomiasis, we developed a PCR to detect *S. haematobium* DNA in serum. We demonstrated that the latter PCR is more sensitive than microscopy when applied on feces and urine, and, when performed on serum, particularly useful to confirm diagnosis during acute urogenital schistosomiasis. We comment on the plausible origin of parasite DNA in relation to the different life cycle stages present in the blood circulation.

## Introduction

Urogenital schistosomiasis due to *Schistosoma haematobium* is a serious underestimated public health problem. It is endemic in 53 countries of the African continent and of the Middle East [1], [2]. Adult worms live in the capillary plexus of the bladder and other parts of the urino-genital system and eggs are excreted in the urine and occasionally found in feces.

Diagnosis of *S. haematobium* infections is traditionally done by microscopy but is often unreliable due to the circadian and day-to-day variations in egg excretion, and to low parasite load, especially in the traveler. Antibody-based assays are useful to confirm infection, but do not distinguish active infection from past exposure, and false-negative results occur, mainly in *S. haematobium* infections. Antibody tests are usually negative during acute symptomatic schistosomiasis. Assays that detect circulating antigens seem very promising in the early phase of infection but still lack sensitivity in the diagnosis of light infections [3], [4], [5], [6].

Recently, we developed a genus-specific real-time PCR (further called ‘genusPCR’) that sensitively detect all human infectious *Schistosoma* species in feces and urine [7]. The genusPCR was not able to detect schistosome DNA in serum although molecular analysis of serum is of interest in acute schistosomiasis before detectable levels of eggs are excreted [8]–[12]. In 2009, Wichmann and colleagues [10] described a real-time PCR, targeting a highly repeated 121-bp sequence of *S. mansoni* (named Sm1-7) to detect cell-free schistosome DNA in serum. This was proven successful in acute and chronic *S. mansoni* infection, but not so much in *S. haematobium* infection. To fill that gap, we developed a real-time PCR specific for the diagnosis of *S. haematobium* in serum samples. The real-time PCR targets *Dra1*, a *S. haematobium*-specific 121-bp repeat sequence originally described by Hamburger *et al.* and present in hundreds to thousands of copies and representing at least 15% of its genome [13]. We first tested this PCR (further called ‘draPCR’) on urine and feces samples to evaluate its species-specificity and its performance in comparison with microscopy, and then on serum samples to determine its potential as diagnostic tool for acute phase schistosomiasis.

## Methods

### Ethics statement

The diagnostic procedures described in this manuscript are part of the standard diagnostic work-up of patients suspected of schistosomiasis. All samples were routine diagnostic samples from patients presenting at the Institute of Tropical Medicine (ITM, Antwerp, Belgium) policlinic and were stored after completion of the routine tests. The ITM has the policy that sample left-overs of patients presenting at the ITM policlinic can be used for research unless the patients explicitly state their objection. The Institutional Review Board of ITM approved the institutional policy of this presumed consent as long as patients' identity is not disclosed to third parties. All data have been analysed anonymously.

### Clinical samples

PCR analysis was retrospectively performed between January and October 2012 on samples that were stored at <−18°C between 2006 and 2012. In total, 330 clinical samples from 187 patients were analysed of which 110 urine, 84 stool, 126 serum, 9 blood samples, and one biopsy sample. The samples were from 145 travelers and 42 migrants that presented at the outpatient clinic of the ITM, Antwerp, Belgium. The patients travelled to Africa (92.5%), Asia (5.4%), the Middle East (1.6%) and South-America (0.5%). The median time interval between return and sample collection was 82 days (varying from 1 day to 1851 days).

Based on laboratory findings, the stool, urine and/or serum samples were selected from patients with confirmed (n = 47) or suspected (n = 140) intestinal or urogenital schistosomiasis. Confirmed cases were defined as patients with eggs of *S. mansoni* or *S. haematobium* as determined by microscopy. Individuals with a confirmed infection were treated with praziquantel after diagnosis. Serum of confirmed cases was collected before or at the time of egg detection, or after treatment. Suspected cases were patients who presented after a stay in an endemic region with clinical symptoms and/or with an increase in eosinophils (>0.45×10^9^/L), a positive serology (IHA titer ≥1/160 or positive ELISA), the presence of Charcot-leyden crystals in feces or hematuria (>7 RBC/µL) or who travelled together with a confirmed case.

### DNA extraction

As previously described [7], [14], DNA was extracted with the QIAamp DNA stool mini kit (Qiagen Benelux, Venlo, The Netherlands) from 1 gram of feces that was dissolved into 5 mL ASL buffer (Qiagen). An 200 µl urine sediment was processed for DNA extraction with the QIAamp DNA mini kit (Qiagen) after centrifugation of 10 mL of urine and three wash/centrifugation steps. For DNA extraction of serum with the QIAamp DNA MIDI kit (Qiagen) or by phenol/chloroform, 1 to 2 mL of serum was used [7].

### Control samples

Positive control DNA of *S. mansoni* and *S. haematobium* were kindly provided by Dr. T. Huyse (ITM/KUL, Belgium). Positive control DNA of *S. intercalatum* and *S. guineensis* were kindly provided by Dr. F. Allan from SCAN at the Natural History Museum (London, UK) [15]. DNA was extracted from one adult worm and used in a 1/100 dilution (∼0.1 ng/µL). Positive control DNA of *S. mekongi* was obtained from a stool sample of a patient seen at ITM, containing 50 eggs per gram (EPG). Positive control DNA of *S. japonicum* derived from cercariae spotted on FTA filter paper kindly provided by Dr. J.P. Webster (London, UK).

### Primer and probe design

The highly repetitive *Dra1* sequence of *S. haematobium* (Accession number DQ157698.1) was selected as target and primers were identical to those described [13] (Sh-FW 5-gatctcacctatcagacgaaac-3′; Sh-RV 5′-tcacaacgatacgaccaac-3′). An additional fluorescent labeled hydrolysis probe was developed (Sh-probe 5′-tgttggtggaagtgcctgtttcgcaa-3′) for real-time monitoring of the PCR signal and was labeled with a 5′-FAM reporter, an internal ZEN quencher and a IowaBlack Fluorescent Quencher at the 3′-end (IDT, Leuven, Belgium). The amplicon size was 96 base pairs.

### Real-time PCR

The draPCR was performed with a 25 µL reaction mix containing 5 µL DNA, 1× Perfecta qPCR Supermix (Quanta Biosciences), 500 nM of Sh-FW and Sh-RV primer, 250 nM of Sh-probe and 0.1 mg/mL bovine serum albumin. The program consisted of an initial step of 2 min at 95°C followed by 50 cycles of 15 s at 95°C and 60 s at 60°C.

The reaction was run on the SmartCycler II (Cepheid Benelux, Belgium). DNA detection was expressed by Cycle threshold (Ct)-values. In every run, the non-template control was negative (Ct = 0) and the *S. haematobium* control was positive.

To detect DNA of schistosome species other than *S. haematobium*, the Sm1-7PCR and genusPCR were performed as described before [7], [10].

### PCR validation

The primer and probe design was verified with Integrated DNA Technology (IDT) Oligo Analyzer software (v3.1) (http://eu.idtdna.com/analyzer/Applications/OligoAnalyzer/). Primer and probe specificity was checked *in silico* by BLAST analysis (http://blast.ncbi.nlm.nih.gov/Blast.cgi) and by 2% agarose gel electrophoresis at 100 V for 35 minutes.

The analytical specificity of the PCR was tested on a panel of clinical control samples containing 23 different intestinal or blood parasites. The panel included stool samples (n = 14) from patients infected with protozoa (*Giardia lamblia, Entamoeba dispar, E. histolytica, Blastocystis hominis, Enterocytozoon bieneusi, Encephalitozoon spp*), nematodes (*Ascaris lumbricoides, Strongyloides stercoralis, Trichostrongylus spp., Trichuris trichiura*, or *Ancylostomidae*), trematodes *(Clonorchis spp., Fasciola hepatica)* or a cestode *(Taenia saginata)* and blood samples (n = 9) from patients infected with *Plasmodium falciparum*, *P. vivax*, *P. ovale*, *P. malariae*, *Leishmania donovani, Loa loa, Onchocerca volvulus, Dirofilaria repens* or *Trypanosoma brucei rhodesiense*.

The detection limit was determined on a 10-fold dilution series of a stool sample containing 580 EPG of *S. haematobium*. It was diluted in a negative stool sample which was dissolved in ASL buffer (Qiagen). DNA was extracted from each dilution and the highest dilution with a positive signal indicated the detection limit.

The variation in Ct-values was determined in a serum sample that was processed 8 times within the same run (repeatability) or 5 reactions that were run at different days (reproducibility). The coefficient of variation (CV, expressed as %) of the Ct-values was calculated.

### Microscopy

Microscopy was performed on a single urine and/or fecal sample per patient at the time of presentation and in some cases, on a single follow-up sample one month after treatment. Diagnosis of schistosomiasis was confirmed when *S. haematobium* or *S. mansoni* eggs were detected in urine and/or feces.

Microscopic examination of urine samples was performed on the sediment of at least 20 mL end-stream urine and of stool samples following a concentration method on 3 grams of feces that had been homogenized in 42 mL of 10% formaldehyde-saline solution [16]. The infection intensity in stool was expressed by the number of EPG. The limit of detection was 10 EPG.

### Serology

A combination of an in-house enzyme-linked immunosorbent assay (ELISA) using *S. mansoni* antigen (mixture of egg and adult worm extract) and an indirect hemagglutination inhibition assay (IHA), using a *S. mansoni* adult worm extract (ELI.H.A. Schistosoma, EliTech MICROBIO, France) with a cut-off at 1/160, were used to detect anti-schistosome antibodies.

## Results

### Primer and probe design

IDT Oligo analysis approved no self- or heterodimerization between the primers and the probe. BLAST analysis with probe and primers indicated 100% query coverage and maximum identity with *S. haematobium*. No species other than *Schistosoma* were *in silico* recognised by the primers and probe. Gel electrophoresis obtained a single band of expected length for the amplicon of *S. haematobium* and no signal for the non-template control.

### PCR validation

To determine the species-specificity, schistosome species of the three complexes were tested with the draPCR, the Sm1-7PCR and the genusPCR. The DNA controls of human species of the *S. haematobium* complex revealed a strong signal with the draPCR (Ct-values ranging from 15.21 to 16.65) and the cattle species *S. bovis* revealed a signal of medium intensity (Ct 29.68). DNA controls of other *Schistosoma* species gave no (*S. japonicum*, *S. mekongi*) or a very weak signal (*S. mansoni*, Ct 41.93) (Table 1). In comparison, the genusPCR easily recognized all species of the three complexes while the Sm1-7PCR detected a strong signal for *S. mansoni* and *S. bovis*, a medium to weak signal for the human species of the *S. haematobium* complex and no signal for species of the *S. japonicum* complex (Table 1).

**Table 1:** Species-specificity of the draPCR in comparison to the Sm1-7PCR and the genusPCR.

complex	species	host	extract from	draPCR	Sm1-7PCR	genusPCR
**S. mansoni complex**	*S. mansoni*	human	adult worm	41.93*	**15.08**	**21.35**
**S. haematobium complex**	*S. haematobium*	human	adult worm	**15.21**	29.44	**20.67**
	*S. intercalatum*	human	adult worm	**16.65**	39.16	**23.64**
	*S. guineensis*	human	adult worm	**15.12**	38.97	**23.26**
	*S. bovis*	cattle	adult worm	29.68	**13.49**	**18.92**
**S. japonicum complex**	*S. japonicum*	human	cercariae	0.00	0.00	**23.52**
	*S. mekongi*	human	clinical sample	0.00	0.00	**27.98**

*Ct-values indicate a strong (Ct<28, highlighted in bold), medium (Ct between 28–38), weak (Ct>38) or no (Ct = 0) recognition of the species.*

* *a 5-fold dilution series on this DNA demonstrated that the weak signal resulted from cross-amplification and not from a-specific background noise.*

Of interest is the difference in Ct-values measured for *S. haematobium* (Ct 15.21) and *S. mansoni* (Ct 41.93) by the draPCR. Since the amount of amplicon doubles every PCR cycle (*i.e.* increase by one log_2_), the difference of 26 Ct's is equivalent to a more than 67 million times lower sensitivity to detect *S. mansoni* in comparison to *S. haematobium*. The same counts for the detection of the *S. bovis* species in comparison to the other *S. haematobium* complex species by the draPCR with a difference of 14 Ct's that accounts for a 16,000 times lower sensitivity (Table 1).

No cross-reaction was seen with the draPCR in the 23 control samples with intestinal and blood parasites other than *Schistosoma*.

The analytical sensitivity demonstrated a detection limit of 0.5 EPG.

Repeatability and reproducibility testing revealed a CV of 1.03% and 1.04% respectively.

### PCR analysis on urine samples and biopsy

A panel of 110 urine samples and one bladder wall biopsy was analysed with the draPCR. A positive PCR signal was obtained in 14 urine samples (Ct-values ranging from 16.77 to 32.40) of which seven were positive for *S. haematobium* ova by microscopy (Table 2). The other seven urine samples (Ct-values ranging from 30.74 to 46.63) were from patients treated for schistosomiasis two weeks or one month before (n = 2) or from patients without previous treatment but with anti-schistosome antibodies (n = 3) or with *S. haematobium* eggs in feces (n = 1) or in a urine sample obtained three days earlier (n = 1). The latter urine sample also contained *Trichomonas vaginalis*. The 96 urine samples that were negative for *S. haematobium* or any other parasite by microscopy, were also negative by PCR (Table 2).

**Table 2:** Evaluation of the draPCR on urine samples.

	Microscopy (urine)		
Dra PCR	*S. haematobium*	negative	Total
positive	**7**	**7**	14
negative		**96**	96
**Total**	7	103	110

The biopsy containing *S. haematobium* eggs was positive by PCR with a Ct-value of 17.08.

### PCR analysis on stool samples

The draPCR was evaluated on a panel of stool samples in which eggs of *S. haematobium* (n = 11), of *S. mansoni* (n = 21) or no schistosome eggs (n = 52) were microscopically detected. All samples with eggs of *S. haematobium* were positive (Ct-values ranging from 20.35 to 37.87) and all samples with eggs of *S. mansoni* were negative (Table 3). In addition, the draPCR revealed a positive signal in three samples without eggs (Ct-values varied from 36.78 to 45.33), two of which were follow-up samples of confirmed patients one month after treatment and one from a clinically suspected patient with eosinophilia.

**Table 3:** Evaluation of the draPCR on feces.

	Microscopy (feces)			
DraPCR	*S. haematobium*	*S. mansoni*	negative	Total
positive	**11**		**3**	14
negative		**21**	**49**	70
**Total**	11	21	52	84

### PCR analysis on serum

There is no reference method for schistosome DNA detection in serum. We therefore used the level of evidence of infection (confirmed *S. haematobium* (n = 12) or *S. mansoni* infection (n = 20) or suspected cases (n = 64)) as a reference (Table 4). Of all suspected cases, 8/64 were migrants and 56/64 travelers of whom serum was collected within 12 weeks upon return in 39 travelers and after more than 12 weeks (varying from 91 to 336 days) in 17 travelers.

**Table 4:** Evaluation of the draPCR on serum.

	Infection status Number of serum samples (number of patients)			
DraPCR	*confirmed S. haematobium*	*confirmed S. mansoni*	Suspected	Total
positive	**22 (11)** $		**5 (2)** ○	27(13)
negative	**1 (1)** *	**22 (20)**	**85 (62)**	108 (83)
**Total**	23 (12)	22 (20)	90 (64)	135 (96)

$ *Of the 22 samples, 11 serum samples were obtained at the moment of egg detection, one sample before egg detection, and 10 samples after treatment. Of the 11 patients with a confirmed S. haematobium infection, 7 patients demonstrated eggs in urine (see* *Table 2* *) and 4 patients had eggs in feces (part of the group described in* *Table 3* *).*

* *only 1 mL of serum was available for PCR analysis and it was obtained 514 days after treatment.*

○ *2 samples were collected before treatment, and 3 samples were taken 21 to 75 days after treatment.*

In total, 135 serum and blood samples were analysed from 96 patients of whom 64 patients with a single serum sample and 32 patients with one or more follow-up samples. No discordant results were obtained in different samples from the same patient.

The draPCR was positive in 27 samples from 13 patients of which 11 patients (22 samples) with a microscopic confirmed *S. haematobium* infection and two patients (5 samples) with a clinical suspicion based on the presence of eosinophilia (0.88 and 2.13 10*9/L) and anti-schistosome antibodies (IHA 1/160 and 1/640) (Table 4). Of all 22 PCR positive samples of individuals with a confirmed *S. haematobium* infection, all serum samples collected at the same date of the parasitological confirmation (n = 11), were positive. All follow-up serum samples obtained 14 to 96 days after treatment (n = 10 from 8 patients), were also positive by PCR but demonstrated higher Ct-values. In two of the 8 patients, both serological tests remained negative 1 month and 2 months after treatment.

PCR was additionally positive on one serum collected about 5 weeks after exposure (n = 1) while at that moment the urine and feces were microscopically negative and no antibodies could be detected. Detection of ova 42 days later, confirmed the *S. haematobium* infection.

In one serum sample from a patient for which a single *S. haematobium* egg was detected in urine 514 days after treatment, no PCR signal was observed but the analysis was performed on an insufficient volume of serum (1 mL instead of 2 mL). All other serum samples with a negative PCR signal were from patients with a confirmed *S. mansoni* infection (20 patients, 22 samples) or from clinically suspected patients without schistosome eggs in urine and feces (62 patients, 85 samples) (Table 4). Four of these suspected patients had microscopically confirmed infections with *Ascaris lumbricoides* (n = 1), *Ancylostomidae* (n = 1), *Strongyloides stercoralis* (n = 1) or *Trichuris trichuria* (n = 1) and nine had been treated 6 months to 3 year prior to serum collection. The 85 PCR negative samples showed schistosomal antibodies by ELISA (n = 4), IHA (n = 10) or both (n = 15) and/or belonged to patients suspected because of recent freshwater exposure or eosinophilia.

## Discussion

Urogenital schistosomiasis remains an important public health problem affecting approximately 112 million people, about half of the worldwide schistosome infections [2]. Schistosomiasis imported by travelers, expatriates and migrants is often caused by *S. haematobium*, with a frequency in the same range to that of *S. mansoni* [6], [17]–[19].

The presently developed PCR was designed to be used as a highly sensitive diagnostic tool for urogenital schistosomiasis in travelers returning from endemic regions. We opted for a real-time PCR format which has a short turn-over time and is preferred over conventional PCR methods due to its lower risk of contamination and higher sensitivity [20], [21]. The latter is of particular interest in travelers as they have often low parasite loads in the acute phase [6], rendering confirmation by microscopy erratic. The draPCR was able to detect all microscopy-confirmed *S. haematobium* infections in urine, bladder wall biopsy and feces and demonstrated no cross-reaction in clinical samples with microscopy-confirmed *S. mansoni* and other intestinal or blood parasites. Moreover, the draPCR detected ten extra *S. haematobium*-positive samples (7 urine and 3 stool samples) from 8 non-egg excretor patients that were highly suspected for urogenital schistosomiasis based on recent freshwater exposure, a strong antibody response (IHA≥1/1280) and/or the presence of eosinophilia. This confirms previous findings that PCR is highly sensitive in urogenital schistosomiasis diagnosis [22]–[24]. The draPCR can be of value on urine and stool samples of suspected patients when no eggs can be demonstrated by microscopy, especially as sampling is not invasive. Alternatively, the genus-specific PCR [7] could be used on urine and feces enabling the detection of schistosome DNA, regardless the causal species.

Besides the excellent performance of the draPCR on urine and stool samples, the most striking result of this study is the specific detection of *S. haematobium* in serum. All but one of the serum samples from patients with a confirmed *S. haematobium* infection, and none of the serum samples from patients with a confirmed *S. mansoni* infection, were positive with the draPCR. Schistosomal DNA could additionally be detected in the serum of one patient about 5 weeks after freshwater exposure and 42 days before confirmation of the *S. haematobium* infection by microscopy. This clearly demonstrates the diagnostic potential of the draPCR to detect *S. haematobium* in serum during the acute phase of the infection.

One serum sample of a patient with a confirmed *S. haematobium* infection was negative, which could be explained by previous treatment or the rather low volume (1 mL) of serum analysed. Due to the retrospective design of this study, PCR could not always be performed on an adequate volume of serum. We consider 2 mL as the optimal volume required for analysis.

Two extra *S. haematobium* infections were detected by the draPCR in serum samples of suspected patients. False-positivity by PCR seems very unlikely as both patients had recent exposure to freshwater in Mali, developed typical severe symptoms related to Katayama syndrome with hypereosinophilia and had a positive serological response five to eight weeks post-exposure. Moreover, *S. haematobium* DNA was also detected in the follow-up serum samples while urine and feces remained negative after treatment. The decreasing PCR signal in follow-up samples demonstrates the PCR's potential to semi-quantitatively monitor treatment. Extra studies are required to confirm this. Further research could additionally compare the persistence of detectable levels of parasite DNA in serum with levels of circulating antigen that are more related to the actual worm burden and rapidly decrease after treatment [25]. Also, more scientific data is needed to assure when parasite DNA is cleared from the blood stream after treatment in order to determine the discriminating power of PCR between active or past present infections with the same *Schistosoma* species. PCR positivity was seen until at least 3 months after treatment in the present study, and up to more than one year after treatment in patients with *S. mansoni* infections [10].

A drawback of this study is that we had no genital samples available to test with the draPCR. *S. haematobium* parasites can cause genital schistosomiasis [1], [26] but in travelers, only few cases were reported [26], [27]. Only one bladder wall biopsy was tested, and although this does not allow drawing conclusions, it is worth to mention that examination of tissue samples by PCR might be helpful to diagnose schistosomiasis in non-egg excretor individuals. Another drawback is that due to the retrospective sample collection, early acute phase samples were not frequently available because antibody or egg detection followed weeks later. A prospective study is needed with selection of patients during the (early) acute phase in order to compare the performance of different tools for urogenital schistosomiasis diagnosis [24].

The PCR targets *Dra1*, a sequence specific for *S. haematobium* [13] and previously successfully used in a conventional PCR on cercaria and infested snails [28] and on urine [24]. Similar to the Sm1-7 121-bp tandem repeat sequence that comprises 11% of the *S. mansoni* genome [8], [10], [29] and to the multicopy *retrotransposon* gene representing 14% of the *S. japonicum* genome [30], *Dra1* also has a highly repetitive nature and represents 15% of the *S. haematobium* genome [13]. The presence of multiple copies of this target sequence enables the highly sensitive detection of *S. haematobium* DNA in serum and probably explains why the single copy 28S gene used in the genusPCR [7] was not successful to detect schistosome DNA in this matrix.

The findings of this study demonstrate that the draPCR for detection of *S. haematobium* infections in serum is complementary to the Sm1-7PCR that is most sensitive to detect *S. mansoni* infections [9]–[10]. Furthermore, we demonstrated the species-specificity of both PCRs in control DNA of adult worm extracts. Apart from the strong signal for the human species of the *S. haematobium* complex group, we also observed a very weak signal for *S. mansoni* with the draPCR and a weak signal for *S. haematobium* with the Sm1-7PCR. This can be explained by the fact that the highly repetitive sequences of *S. haematobium* or *S. mansoni* respectively, are most likely present in at least a single copy in the genome of other *Schistosoma* species [13] and are only detectable when a huge amount of parasite DNA is present as in the case of the adult worm extracts. Since we did not detect a signal with the draPCR in all 43 clinical samples of patients with confirmed *S. mansoni* infections (egg load varying between 10 and 120 EPG), we conclude that the draPCR and Sm1-7PCR are suitable for analysis of serum of patients suspected for urogenital and intestinal schistosomiasis, respectively.

What do we detect by the draPCR in serum, urine and feces?

In analogy with prenatal diagnostics and oncology [31], [32], Wichmann *et al* [10] used the term ‘cell-free parasite DNA’ (CFPD) to comprise the DNA that was detected in serum. Indeed, due to the high parasite turnover, diverse stadia of the parasite might be present in the blood circulation and are detectable depending on the phase of the infection. Once penetrated through the human skin, schistosomules travel with the venous circulation to the lungs within 7 to 10 days and thereafter to the liver region for maturation [1]. In the acute phase of urogenital infections, the draPCR probably detects DNA of degrading schistosomules or juvenile worms that did not survive or mate. Schistosomes are complex multicellular eukaryotes, and the schistosome DNA in serum might also originate from rapid turn-over of the tegument during maturation of the worm [33]. In addition, after the upstream migration of mature worms to the venous plexus of the bladder and deposition of eggs, DNA of eggs that circulate into the systemic circulation due to retrograde venous flow could be detected. In chronic infections, DNA from desintegrated eggs, or from killed worms after treatment could also be a target for PCR in serum.

In urine samples, the PCR primarily detects DNA from *S. haematobium* eggs. It is not unlikely that also transrenal nucleic acids of breakdown products of the parasite are detectable in the urine as demonstrated before for *S. mansoni* [34], [35] and other parasitic infections [36], [37]. In feces, DNA of *S. haematobium* eggs can be found due to the atypical location of the worm in the colon or rectal wall, or due to contamination of the stools with urine in case of a high-intensity infection [1], [38].

So far, no *S. haematobium*-specific PCR has been described before to be used in human serum of recently infected travelers. Our findings suggest that the draPCR in serum is suitable for diagnosis of urogenital schistosomiasis in a non-endemic setting and might be of value in diagnosing travelers during the acute phase of infection (4 to 6 weeks after exposure to infested water) before eggs excretion and seroconversion, and in light infections. Serology tests turn positive only about 6 to 12 weeks after exposure [6]. In addition, weak positive serological reactions are difficult to interpret and false-negative tests occur, especially with *S. haematobium* [4], [39], [40]. Further prospective evaluation of the draPCR on serum samples is needed, to demonstrate its diagnostic role during the early acute phase of the infection.

## Supporting Information

Supporting Information S1 **STARD checklist.**
(DOC)

Figure S1 **Flowchart for evaluation of the draPCR.**
(DOC)
