# Supplementary material for: A Schistosoma haematobium-Specific Real-Time PCR for Diagnosis of Urogenital Schistosomiasis in Serum Samples of International Travelers and Migrants
Source: PLoS Negl Trop Dis. 2013 Aug 29;7(8):e2413. doi: 10.1371/journal.pntd.0002413 (PMC3757062; doi:10.1371/journal.pntd.0002413)
Supplement: Figure S1 — Flowchart for evaluation of the draPCR. (DOC) [file pntd.0002413.s002.doc]

# Supporting Figure S1 - Flow chart for evaluation of the draPCR

Retrospective selection of samples of patients returning from an endemic region with confirmed or suspected schistosomiasis based on laboratory data or with clinical symptoms or traveling together with a confirmed case

Excluded due to unavailability of the sample (*e.g.* not stored)

Testing of the samples with draPCR

n=330

Urine

(n=110)

Bladder wall biopsy (n=1)

Feces

(n=84)

Serum

(n=135)

Positive by microscopy and PCR

(n=7)

Negative microscopy and PCR

(n= 96)

Positive by microscopy and PCR

(n=1)

Positive by microscopy and PCR

(n=11)

Confirmed infection

n=23 (Sh) n=22 (Sm)

(microscopy on feces/urine positive)

Suspected infection n=90

(microscopy

negative)

Sh negative microscopy and PCR

(n= 70)

Positive by PCR and negative by microscopy

(n=7)

Positive by PCR and negative by microscopy

(n=3)

Positive by PCR

(n=22)

Sh

Negative by PCR

(n=22)

Sm

Positive by PCR

(n=5)

Negative by PCR

(n=85)

Negative by PCR

(n=1)

Sh

7 extra positive urine samples with PCR compared to microscopy

*Supportive clinical, epidemiological and laboratory data*

3 extra positive fecal samples with PCR compared to microscopy

1 confirmed infection missed by PCR

5 extra positive serum samples of 2 patients with PCR
